# Editorial: Insights in genetics of common and rare diseases 2024–2025

**DOI:** 10.3389/fgene.2026.1996785

**Published:** 2026-10-27

**Authors:** Jordi Pérez-Tur, Mara Marongiu, Erica E. Davis

**Affiliations:** 1 Unitat de Genètica Molecular, Institut de Biomedicina de València-CSIC, Valencia, Spain; 2 Centro de Investigación Biomédica en Red sobre enfermedades Neurodegenerativas (CIBERNED), ISCIII, Madrid, Spain; 3 Istituto di Ricerca Genetica e Biomedica, Consiglio Nazionale delle Ricerche (CNR), Lanusei, Italy; 4 Stanley Manne Children’s Research Institute, Ann & Robert H. Lurie Children’s Hospital of Chicago, Chicago, IL, United States; 5 Department of Pediatrics and Department of Cell and Developmental Biology, Feinberg School of Medicine, Northwestern University, Chicago, IL, United States

**Keywords:** precision diagnostics, rare disease discovery, genotype-phenotype correlation, translational biology, genomic medicine, prenatal and developmental genetics

In the rapidly evolving landscape of genetic research, particularly within the realms of common and rare diseases, the past decade has seen remarkable achievements and breakthroughs. Fueled in large part by technical advances, the subspecialties of functional genomics, bioinformatics and precision medicine have led to exciting new insights. These developments have heralded the discovery of new disease genes; pathogenic mechanisms that often translate into major improvements in diagnosis; prognostic stratification and therapeutic approaches in diverse rare and common genetic disorders. In this Research Topic, we highlight manuscripts that reflect the emergent metamorphosis in the genetics of common and rare diseases.

This manuscript Research Topic can be divided into three major partially overlapping themes (Figure 1): (1) Genomic medicine, with manuscripts pertaining to diagnostics (Zhang et al.; Malmgren et al.; Fang et al.; Fang et al.; Lan et al.), population genomics (Gui et al.; Mouawad et al.), genotype-phenotype description (Xu et al.; Gupta et al.; Bi et al.; Feghali et al.; Orozco et al.; Wei et al.; Chan et al.), prenatal and developmental genetics (Xu et al.; Zhao et al.; Yin et al.; Wang et al.; Cai et al.) or complex disease genetics (Zhu et al.; Ji et al.; Menon et al.; Menon et al.); (2) rare disease discovery with papers grouped in genotype-phenotype description (Xu et al.; Gupta et al.; Bi et al.; Feghali et al.; Orozco et al.; Wei et al.; Chan et al.), prenatal and developmental genetics (Xu et al.; Zhao et al.; Yin et al.; Wang et al.; Cai et al.), congenital heart disease and copy number variants (CNVs) (Fifirig et al.) and novel disease gene reports (Lucia-Campos et al.; Yang et al.; Xin et al.; Ma et al.; Lin et al.); (3) mechanisms of translational biology, with papers also clustering in prenatal and developmental genetics (Xu et al.; Zhao et al.; Yin et al.; Wang et al.; Cai et al.), complex disease genetics (Zhu et al.; Ji et al.), congenital heart disease and CNVs (Fifirig et al.), descriptions of novel genes (Lin et al.; Ma et al.) and functional genomics (Zhang et al.; Li et al.; Na et al.).

**FIGURE 1:**
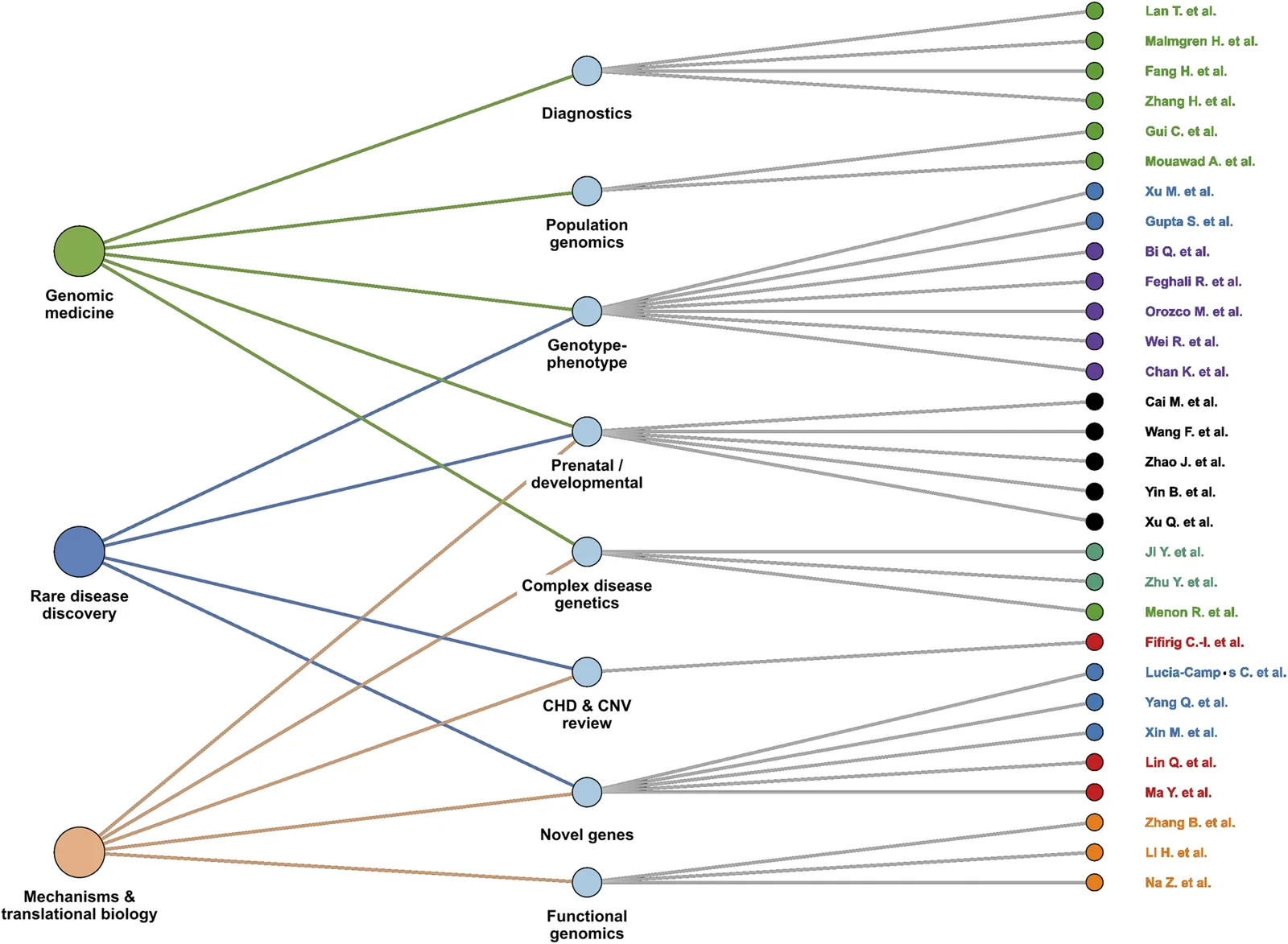
Network representation of the manuscripts included in the Research Topic *Insights in Genetics of Common and Rare Diseases 2024–2025*. Individual manuscripts are connected to scientific clusters, which are subsequently linked to three overarching themes: Genomic Medicine and Precision Diagnostics (green, theme 1), Rare Disease Discovery and Clinical Characterization (blue, theme 2), and Disease Mechanisms and Translational Biology (orange, theme 3). Manuscript node colors indicate thematic membership: green (theme 1), blue (theme 2), orange (theme 3), purple (themes 1 and 2), red (themes 2 and 3), teal (themes 1 and 3), and black (all three themes). Cluster nodes are shown in light blue, while theme nodes are colored according to the corresponding overarching theme. Connections between manuscripts and clusters represent the primary scientific grouping of each contribution, whereas connections between clusters and themes illustrate broader conceptual relationships across the Research Topic.

The diagnostics cluster of the genomic medicine theme illustrates an increasing integration of genomic technologies into routine medical practice. Lan, T. and coworkers employed long-read sequencing to substantially improve detection and characterization of *CYP21A2* variants in 21-hydroxylase deficiency (21-OHD; MIM #201910) compared to traditionally-employed multiplex ligation-dependent probe amplification (MLPA) plus long-PCR sequencing, and showed that a next-generation sequencing (NGS)-based technique is also an efficient approach to diagnose 21-OHD patients (Lan et al.). Likewise, Malmgren, H. and collaborators assessed the diagnostic yield in individuals affected with a variety of clinical symptoms using parent-proband trio exome and genome sequencing (ES/GS). This work provides evidence of the clinical utility of comprehensive genomic testing. Further, the authors reinforce how trio analysis has a higher diagnostic yield compared to singleton analysis (Malmgren et al.). Fang, H. and colleagues performed an observational study analyzing the association of a mitochondrial DNA variant with mitochondrial diabetes mellitus (MDM; MIM #520000). In particular, they demonstrate that a minimally invasive Research Topic sampling technique was effective for obtaining patient samples suitable for diagnosis (Fang et al.). To determine the incidence and the genetic basis of inborn errors of metabolism, Zhang, H. and coworkers used tandem mass spectrometry and molecular testing on a population of more than 1.4 million newborns in Xinjiang (China) (Zhang et al.). Large scale screening was also employed by Gui, C. and collaborators to identify rare hemoglobin variants Hb O-Arab and Hb D-Punjab in Guangxi (China), expanding the known hemoglobinopathy spectrum across diverse ancestries in China (Gui et al.). A population-based study was also leveraged by Mouawad, A. and colleagues who analyzed a Lebanese cohort and confirmed the association of a promoter variant in *MUC5B* with idiopathic pulmonary fibrosis (MIM #178500) (Mouawad et al.). Further, in a large South-Asian population, Menon, R. and coworkers validated the utility of a polygenic risk score for body-mass index as a predictor of risk for obesity (Menon et al.).

Taken together, these advances demonstrate the increasing integration of genomic technologies into routine medical practice. Moreover, the collective output shows how diagnostic approaches are being reshaped across distinct clinical settings and how the field is moving towards a predictive era for complex diseases. Notably, population genomics and molecular epidemiology have closed knowledge gaps of specific rare conditions in large cohorts.

A substantial recent trend is the refinement of genotype-phenotype relationships, in both genomic medicine and rare disease discovery. Bi, Q. and colleagues showed that individuals carrying pathogenic variants in *PCDH15* (MIM #605514) had marked benefits from cochlear implantation regardless of the existing phenotype: isolated deafness or Usher syndrome (Bi et al.). Additionally, Feghali, R. and coworkers characterized *MEFV* variants (MIM #608107) in familial Mediterranean fever (MIM #249100) in a Lebanese population (Feghali et al.) and defined the genetic landscape of this disease in different geographic subpopulations. Similarly, Orozco, M. and collaborators characterized variants linked to Duchenne muscular dystrophy (MIM #310200) in Guatemala, emphasizing the need for easy and fast access to genetic infrastructure. Exemplifying a rare inherited multisystem disorder, Shwachman-Diamond syndrome (MIM #260400 and #617941), Wei, R. and colleagues reported clinical heterogeneity in the phenotype including pancreatic insufficiency, neutropenia, developmental anomalies and neurocognitive manifestations (Wei et al.). Further demonstrating the predictive power of the genotype, Chan, K. and coworkers studied a cohort of rare forms of congenital adrenal hyperplasia combining clinical observations and genetic analysis to demonstrate that genomic characterization is instrumental in defining subtypes of clinically overlapping endocrine disorders while also describing novel variants in steroidogenic genes (Chan et al.). Accordingly, Xu, M. and collaborators showed for individuals with Netherton syndrome (MIM #256500) that the distribution of *SPINK5* variants relates to disease severity, including infant mortality, nutritional status, as well as dermatological manifestations (Xu et al.). In a related study, Gupta, S. and colleagues probed the spectrum of RASopathies in a large North-Indian cohort. They found a previously undescribed correlation between specific molecular defects and clinical presentations (Gupta et al.). Collectively, these studies emphasize the important role that genetic analysis of clinically characterized samples plays in moving from mere diagnosis or confirmation of disease etiology, to informing patients of their prognosis, therapeutic choices and genetic counseling.

This Research Topic also illustrates the rapid integration of computational biology and systems-level approaches and their growing impact in human genetics. Ji, Y. and coworkers suggested shared or overlapping molecular mechanisms of immune dysregulation and inflammation in polycystic ovary syndrome (MIM #184700; now known as polyendocrine metabolic ovarian syndrome, PMOS) and rheumatoid arthritis (MIM #180300). By analyzing available datasets in both conditions, they found subsets of shared genes with dysregulated expression involved in cell death, inflammation and redox pathways. Through a large meta-analysis, Zhu, Y. and collaborators refined the relationship between the *TGF-β1* rs1982073 variant and susceptibility to autoimmune disease, especially in Asian populations (Zhu et al.). These papers demonstrate how sophisticated computational approaches help delineate biological connections between apparently unrelated diseases, while concomitantly identifying potentially promising therapeutic targets. In a similar approach applied to congenital heart disease associated with the 15q11.2 microdeletion (MIM #615656), Fifirig, C.-I. and colleagues summarized the mechanistic evidence linking the 15q11.2 BP1-BP2 microdeletion and the four impacted genes: *TUBGCP5, CYFIP1, NIPA1* and *NIPA2*, in phenotype manifestation. The authors showed that the prevalence of this condition in deletion carriers ranges from 10% to 30%, possibly reflecting its low penetrance and variable expressivity (Fifirig et al.).

One of the most prominent themes is the expansion of disease-associated genes and genotype-phenotype relationships. For example, Lucia-Campos, C. and coworkers reported an association between an intragenic duplication in *AFF2* and a Cornelia de Lange syndrome-like phenotype. This extends the group of transcriptional regulators implicated in this family of disorders to *AFF2* (Lucia-Campos et al.). Similarly, Yang, Q. and collaborators described the familial involvement of *UBAP2L* in a recently recognized condition showing a neurodevelopmental disorder (MIM #620494) with developmental delay, impaired language, behavioral abnormalities, seizures and dysmorphic facies (Yang et al.). The link with developmental and connective tissue disorders is also the subject of work published by Xin M. and colleagues, who identified a pathogenic *COL2A1* variant associated with acetabular dysplasia (Xin et al.), and of Lin, Q. and coworkers who found pathogenic or likely pathogenic variants in *PLOD1*, *COL1A1*, *COL5A2* and *COL4A1* in four families affected with keratoconus (MIM #148300) (Lin et al.). Similarly, through analysis of a zebrafish model, Ma, Y. and collaborators found a previously unrecognized role of *MFSD3* in auditory development and sensory hair-cell maintenance (Ma et al.). Collectively, these studies reflect the challenges associated with genetic heterogeneity and the importance of thorough clinical and genetic investigations in unresolved cases. Moreover, these papers support the growing capacity of the field not only to identify disease genes, but also to impact patient management.

While genetic discovery is a critical foundation toward understanding mechanisms, some papers deepened our understanding of the molecular and cellular pathology underlying disease with functional studies. In one example, Zhang B. and colleagues showed that *CFTR* variants may exert their pathogenic effects by altering RNA splicing rather than through an altered protein-dependent mechanism (Zhang et al.). Li, H. and coworkers showed that a marked proportion of variants in *COL4A5* induced aberrant transcripts and are usually associated with more severe renal outcomes (Li et al.). On the other hand, Na X. and collaborators utilized transcriptomic analysis and functional validation to implicate *AURKA*, *BUB1*, *CDK1*, *RAD51* and *TOP2A* in biliary atresia pathogenesis (Na et al.). These manuscripts highlight a strengthened interest and capacity to move from identification of genetic variants to functional validation to improve mechanistic understanding.

Finally, we note a broad-reaching group of manuscripts span all three main themes: prenatal and developmental genetics. First, in fetuses with hyperechoic kidneys, Cai, M. and colleagues showed the utility of combining ES and microarray analysis to inform choices in pregnancy management (Cai et al.). Similarly, Wang, F. and coworkers found highly variable prenatal phenotypes together with incomplete penetrance in fetuses with recurrent 1q21.1;CNVs exemplifying the challenges of prenatal diagnosis and genetic counselling. In addition to describing new or underreported phenotypes, they report some phenotypes commonly related to 1q21.1 deletion syndrome (MIM #612474) as well as other symptoms more frequently seen in 1q21.1 duplication syndrome (MIM #612475) (Wang et al.). Additionally, Zhao, J. and collaborators described a multigenerational family carrying a balanced t(5; 9)(p15; p24) translocation that resulted in unbalanced chromosomal rearrangements and Cri-du-chat syndrome in some offspring (MIM #123450) (Zhao et al.). This study applied cytogenetic and genomic analysis and may have high clinical relevance for reproductive counseling, risk assessment or genetic diagnosis. In a separate study that sought to identify mechanistic insights in non-syndromic cleft lip, Yin, B. and colleagues identified *SLC23A2* as a significant genetic risk factor supported by experimental evidence to implicate increasing oxidative stress and apoptosis as a possible mechanistic underpinning in both oral epithelial cells and zebrafish models. Thus, they demonstrated an interaction between SLC23A2 function, oxidative stress and ascorbic acid availability (Yin et al.). Finally, the study by Xu, Q. and coworkers on neonatal-onset methylmalonic acidemia (MMA) showed that the disease presented with non-specific symptoms, highlighting diagnostic challenges that can delay disease-specific care. In the small cohort analyzed, most affected individuals had recurrent variants in *MMACHC* and *MMUT*. These studies reinforce the importance of combining genetic and functional analysis for the timely diagnosis of metabolic disorders.

The repertoire of studies in this Research Topic offers a snapshot of the remarkable advances currently taking place in human genetics. New disease genes continue to be discovered, genomic technologies increasingly influence everyday clinical practice, and more refined genotype-phenotype correlations help to improve diagnosis, prognosis, and patient management. The breadth of Research Topic covered reflects the diversity of contemporary genetics research, encompassing rare endocrine and metabolic disorders, developmental syndromes, kidney diseases, pulmonary fibrosis, hearing impairment, and complex multifactorial conditions. A recurring theme throughout the Research Topic is the growing integration of complementary methodologies, including second- and third-generation sequencing, transcriptomics, bioinformatics, newborn screening, and population genetics. Alongside these approaches, functional and experimental studies provide valuable insights into the biological mechanisms that underlie disease, while cohort-based investigations continue to broaden our understanding of genetic variation and its impact on human health across different populations.

Several contributions also point to the direction in which the field is moving. The distinction between rare and common diseases is increasingly blurred, not only because similar genomic, analytical, and functional approaches are now being applied across both areas, but also because these technologies are progressively being incorporated into routine clinical care. At the same time, the widespread availability of genomic tools is encouraging a shift away from the simple identification of genetic variants toward more integrated strategies that combine multiple layers of evidence. By bringing together genomic, transcriptomic, functional, and clinical data, these approaches provide a comprehensive view of disease and support more informed clinical decision-making.

The contributions assembled in this Research Topic underscore several key priorities for advancing human genetics, including the transition from variant discovery to the functional characterization of genetic variation, the integration of genomic and multi-omic data to refine disease mechanisms and support clinical implementation, the expansion of research efforts to encompass more diverse populations, and the increasing recognition of shared genetic architectures across rare and common diseases. Together, these developments reflect the field’s evolution toward a more integrative, mechanistically informed, and translationally oriented paradigm.

Thus, this Research Topic represents more than a snapshot of the state of the art in the genetics of common and rare diseases. Collectively, these studies illustrate how genetic discoveries continue to deepen our understanding of human disease while bringing the promise of precision medicine ever closer to everyday clinical practice.

